# Supplementary material for: Unmet Health Needs among Young Adults with Cerebral Palsy in Ireland: A Cross-Sectional Study
Source: J Clin Med. 2022 Aug 18;11(16):4847. doi: 10.3390/jcm11164847 (PMC9410409; doi:10.3390/jcm11164847)
Supplement: Supplementary file 1 [file jcm-11-04847-s001.zip › jcm-1747138-supplementary.pdf]

Table S1 Associations between intellectual disability, ambulatory status, discharge status and unmet need for speech, epilepsy, equipment, mobility and control of movement

	Speech (n=25)		Epilepsy (n=14)		Equipment (n=51)		Mobility (n=56)		Control of movement (n=48)	
	β (95% CI)	*p*	β (95% CI)	*p*	β (95% CI)	*p*	β (95% CI)	*p*	β (95% CI)	*p*
Unadjusted analyses										
**Intellectual disability**	0.80 (0.16, 4.12)	0.790	1.88 (0.20, 17.3)	0.579	1.40 (0.43, 4.51)	0.573	**3.64 (1.10, 12.0)**	**0.034**	1.00 (0.29, 3.45)	1.000
**GMFCS level IV-V**[a]	**6.00 (1.00, 35.9)**	**0.050**	4.50 (0.34, 60.2)	0.256	1.82 (0.58, 5.70)	0.303	2.75 (0.90, 8.40)	0.076	1.80 (0.55, 5.89)	0.334
**Discharged**	1.56 (0.28, 8.53)	0.611	1.00 (0.12, 8.31)	1.000	0.63 (0.20, 1.97)	0.428	1.78 (0.60, 5.30)	0.302	1.75 (0.52, 5.89)	0.366
Adjusted analysis										
**Intellectual disability**	Not calculated		Not calculated		0.97 (0.25, 3.77)	0.967	3.08 (0.78-12.2)	0.108	0.75 (0.17, 3.37)	0.711
**GMFCS level IV-V**[a]	Not calculated		Not calculated		2.08 (0.54, 8.01)	0.287	1.52 (0.40, 5.68)	0.537	1.90 (0.45, 8.01)	0.381
**Discharged**	Not calculated		Not calculated		0.55 (0.17, 1.80)	0.319	1.73 (0.52, 5.76)	0.368	1.49 (0.41, 5.42)	0.547

[a]Reference: levels I-III

Table S2 Associations between intellectual disability, ambulatory status, discharge status and unmet need for bone or joint problems, positioning, curvature of back, eyesight and pain.

	Bone or joint problems (n=39)		Positioning (n=37)		Curvature of back (n=30)		Eyesight (n=38)		Pain (n=34)	
	**β (95% CI)**	***p***	**β (95% CI)**	***p***	**β (95% CI)**	***p***	**β (95% CI)**	***p***	**β (95% CI)**	***p***
Unadjusted analyses										
**Intellectual disability**	1.11 (0.30, 4.17)	0.876	3.31 (0.76, 14.4)	0.111	1.67 (0.35, 8.04)	0.525	1.42 (0.34, 5.87)	0.631	0.94 (0.14, 6.54)	0.954
**GMFCS level IV-V**[a]	0.74 (0.20, 2.70)	0.649	7.33 (0.81, 66.1)	0.076	0.63 (0.13, 3.09)	0.564	**12.9 (2.22, 74.5)**	**0.004**	0.54 (0.08, 3.74)	0.534
**Discharged**	1.69 (0.44, 6.47)	0.442	1.20 (0.29, 4.94)	0.800	3.18 (0.53, 19.1)	0.205	2.13 (0.46, 9.84)	0.331	0.62 (0.09, 4.29)	0.630
Adjusted analysis										
**Intellectual disability**	1.49 (0.32, 6.93)	0.610	2.14 (0.44, 10.5)	0.349	3.11 (0.50, 19.18)	0.222	Not calculable		1.46 (0.12, 17.6)	0.767
**GMFCS level IV-V**[a]	0.53 (0.11, 2.48)	0.418	5.65 (0.55, 57.7)	0.144	0.23 (0.03, 1.81)	0.162	Not calculable		0.47 (0.03, 6.53)	0.576
**Discharged**	1.97 (0.48, 8.06)	0.345	0.94 (0.19, 4.49)	0.934	7.64 (0.82, 71.4)	0.075	Not calculable		0.81 (0.10, 6.79)	0.842

[a]Reference: levels I-III
